# IL-17F induces inflammation, dysfunction and cell death in mouse islets

**DOI:** 10.1038/s41598-020-69805-2

**Published:** 2020-08-04

**Authors:** Tara Catterall, Stacey Fynch, Thomas W. H. Kay, Helen E. Thomas, Andrew P. R. Sutherland

**Affiliations:** 1 St. Vincent’s Institute of Medical Research, 9 Princes Street, Fitzroy, Melbourne, VIC 3065 Australia; 2 Department of Medicine, St. Vincent’s Hospital, The University of Melbourne, Fitzroy, VIC Australia

**Keywords:** Apoptosis, Interleukins, Chronic inflammation, Type 1 diabetes

## Abstract

Type 17 immune responses, typified by the production of the cytokines IL-17A and IL-17F, have been implicated in the development of type 1 diabetes in animal models and human patients, however the underlying pathogenic mechanisms have not been clearly elucidated. While previous studies show that IL-17A enhances inflammatory gene expression and cell death in mouse β-cells and human islets, the function of IL-17F in pancreatic β-cells is completely untested to date. Here we show that IL-17F exhibits potent pathogenic effects in mouse β-cell lines and islets. IL-17F signals via the IL-17RA and -RC subunits in β-cells and in combination with other inflammatory cytokines induces expression of chemokine transcripts, suppresses the expression of β-cell identity genes and impairs glucose stimulated insulin secretion. Further IL-17F induces cell death in primary mouse islets. This occurs via Jnk, p38 and NF-κB dependent induction of *Nos2* and is completely ablated in the presence of an inducible nitric oxide synthase (iNOS) inhibitor. Together these data indicate that IL-17F possesses similar pathogenic activities to IL-17A in mouse β-cell lines and islets and is likely to be a type 17 associated pathogenic factor in type 1 diabetes.

## Introduction

In type 1 diabetes autoreactive T cells destroy insulin-producing β-cells in the pancreatic islets^1,2^. CD4^+^ and CD8^+^ T cells are the main drivers of disease and secrete high levels of inflammatory cytokines within islets. Exposure of islets to cytokines, such as TNFα, IFNγ and IL-1β, leads to the activation of inflammatory signalling and the production of inflammatory mediators and chemotactic factors such as chemokines. Recent studies indicate that inflammatory cytokines can induce β-cell dysfunction, typified by reduced expression of β-cell identity genes and insulin secretion^3^. β-cell populations exhibiting these characteristics have recently been identified in non-obese diabetic (NOD) mice^4^, suggesting a role in type 1 diabetes pathogenesis. Chronic exposure of islets to inflammatory cytokines has direct pathogenic effects on pancreatic β-cells via the activation of apoptotic cell death pathways^5^. Under these circumstances β-cells can upregulate the expression of inducible nitric oxide synthase (*Nos2*, iNOS) and the resultant production of nitric oxide (NO) leads to apoptotic cell death^5^. In the NOD model and human type 1 diabetic patients, cytokines including IFNs, IL-1β, TNFα, IL-6, IL-12, IL-17A are present in islets during disease course and are likely modulators of important disease processes^6,7^.

Type 17 immune cells, including Th17 cells and γδ T cells, express high levels of the cytokines IL-17A and IL-17F under the control of the RORγt/RORα transcriptional complex^8,9^. Evidence that type 17 immune cells and IL-17A are important contributors to type 1 diabetes pathogenesis comes from several sources^10^. Type 1 diabetic patients have increased production of IL-17A and increased frequencies of IL-17A producing immune cells^11–15^. A Th17 gene signature was observed in the islets of a patient who died soon after diagnosis of type 1 diabetes^15^. Th17 cells can promote type 1 diabetes in NOD mice^16–19^ and anti-IL-17A antibody treatment protects NOD mice from type 1 diabetes^20^. NOD mice treated with a RORγt/RORα inverse agonists have suppressed IL-17A production and are protected from type 1 diabetes^21^. In contrast, IL-17A knockout NOD mice^22^ and IL-17A knockdown NOD mice^23^ have essentially normal type 1 diabetes incidence and timecourse. Thus type 17 immune responses can be pathogenic in type 1 diabetes, however it is likely that IL-17A is not solely responsible for this pathogenicity.

IL-17F and IL-17A are closely related cytokines that exist as homodimers and IL-17A:IL-17F heterodimers^24^. These ligands engage the IL-17RA and -RC receptor complex^24,25^ leading to activation of inflammatory pathways including NF-κB and AP-1 and expression of inflammatory gene products^24^. While IL-17F and IL-17A exhibit similar activities in models of autoimmunity and pathogen clearance including experimental autoimmune encephalomyelitis (EAE) and *Citrobacter rodentium* infection^26,27^, their functions diverge in models of airway inflammation^25^ and colitis^28^ suggesting that the functions of IL-17F and IL-17A are likely to be tissue- and disease model-specific. In contrast to IL-17A, the role of IL-17F in type 1 diabetes pathogenesis is largely unstudied. The sparse evidence to date indicates that IL-17F expression is increased in parallel with IL-17A in the pancreas of NOD mice at diabetes onset^17^, RORγt/RORα inverse agonists also suppress IL-17F production in NOD mice^21^ and circulating IL-17F levels were increased in newly diagnosed type 1 diabetic patients^29^. These data suggest that IL-17F may have pathogenic functions in the context of type 1 diabetes that are yet to be defined. Thus our aim was to determine whether IL-17F exerts pathogenic activities in mouse pancreatic β-cells and islets.

## Research design and methods

### Mice

All animal care and experiments were approved by the St. Vincent’s Animal Ethics Committee. All animal studies were conducted following the guidelines of the institutional animal ethics committee and the experiments were carried out in accordance with the approved guidelines. NOD/Lt and C57Bl/6 mice were bred and housed in microisolator cages under specific pathogen-free conditions at the BioResources Centre.

### Cell culture and CRISPR/Cas9 gene editing

Insulin producing cell lines, NIT-1 and Min6^30^ (a kind gift from Dr. Jun-ichi Miyazaki), were cultured in high glucose Dulbecco's Modified Eagle's Medium (DMEM, Life Technologies) supplemented with 10% heat inactivated fetal calf serum (FCS). CRISPR/Cas9 gene inactivation was performed as previously described^31^ using pLENTICRISPRv2, a gift from Feng Zhang (Addgene plasmid #52961), and primers listed in Supplementary Table 1.

### Islet isolation and glucose stimulated insulin secretion

Mouse islets were isolated as previously described^32^ from 4–6 week old male mice and cultured in Connaught Medical Research Laboratories cell culture media (CMRL, Life Technologies) supplemented with 10% heat inactivated fetal calf serum. Glucose stimulated insulin secretion assays were performed as previously described^33^ except insulin was quantified using a rat/mouse insulin ELISA assay (Millipore).

### Cytokines and inhibitors

Mouse cytokines were used at the following concentrations: mTNFα (In Vitro Technologies) at 100 ng/ml, mIFNγ at 10 ng/ml (Australian Biosearch), hIL-1β at 50 ng/ml (Peprotech), mIL-17A at 25 ng/ml (R&D Systems) and mIL-17F at 100 ng/ml (R&D Systems). Jnk inhibitor (SP600125, Santa Cruz Biotechnology) was used at 50 μM, p38 inhibitor (SB203580, Santa Cruz Biotechnology) was used at 20 μM, NF-κB inhibitor (BAY11-7082, Sigma Aldrich) was used at 10 μM and iNOS inhibitor (*N*-methylarginine, NMMA, Sigma Aldrich) was used at 1 mM.

### RNA extraction, cDNA synthesis and quantitative RT-PCR

Total RNA was isolated with Bioline Isolate II RNA Micro Kit. 1 μg total RNA was used to reverse transcribe using the High Capacity cDNA Reverse Transcription Kit (Applied Biosystems). Quantitative RT-PCR was performed using the AmpiTaq Gold Kit (Applied Biosystems) and Taqman probes with the 7500 Fast Real-Time PCR System (Applied Biosystems). Changes in transcript expression were quantified using the ddCT method using β-actin as an internal control. The list of primers is provided in Supplementary Table 2.

### Flow cytometry and analysis of cell death

Flow cytometry was performed as previously described^34^. IL-17RA antibody was purchased from eBioscience (clone #PAJ-17R) and IL-17RC and IL-17RD biotinylated polyclonal antibodies were purchased from R&D Systems. Cell viability was evaluated by Nicoletti DNA fragmentation analysis^35^. Data was collected on a BD Fortessa flow cytometer (BD Biosciences, San Jose, CA) and subsequently analysed on FlowJo software (version 8.7.3).

### Statistical analysis

Data are presented as the mean ± SEM. Statistical significance was determined using One-Way and Two-Way ANOVA with Bonferonni’s post-test, significance values indicated: p < 0.05 (*), p < 0.01 (**) and p < 0.001 (***). Analysis was performed using GraphPad Prism 8.0 Software.

## Results

### IL-17F induces *Nfkbiz* expression via IL-17RA and -RC in pancreatic β-cells

**Figure 1:**
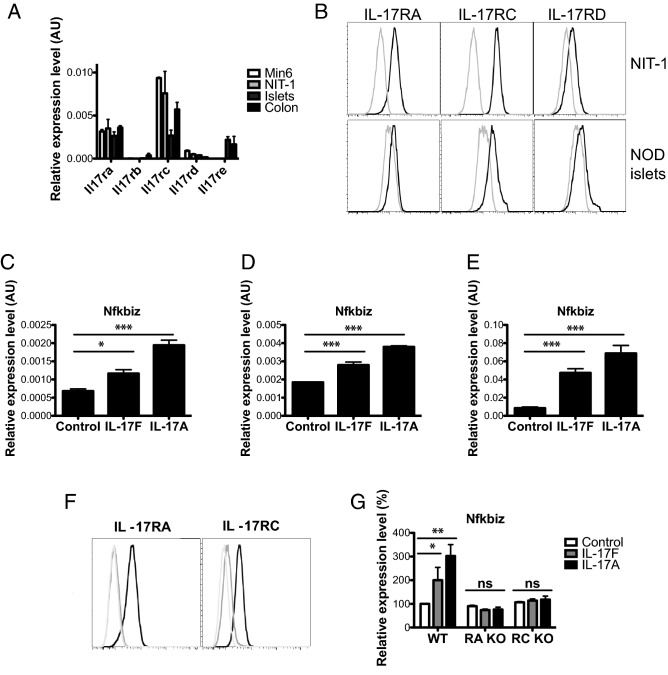
Pancreatic β-cells respond to IL-17F signals via IL-17RA and -RC. (**A**) Total RNA was isolated from Min6, NIT-1, pancreatic islets and colon tissue and the expression of *Il17ra-e* measured by Taqman quantitative RT-PCR. (**B**) NIT-1 cells and primary NOD islets were stained with antibodies against IL-17RA, RC and RD and cell surface expression quantified using flow cytometry (black line = antibody stained, grey line = unstained control). (**C**) NIT-1 cells were stimulated with IL-17F or IL-17A for 2 h and *Nfkbiz* expression measured by Taqman quantitative RT-PCR (n = 4 for all groups), p < 0.05 (*), p < 0.001 (***) One-Way ANOVA with Bonferonni’s Multiple Comparison test. (**D**) Min6 cells were stimulated with IL-17F or IL-17A for 4 h and *Nfkbiz* expression measured by Taqman quantitative RT-PCR (n = 4 for all groups), p < 0.001 (***) One-Way ANOVA with Bonferonni’s Multiple Comparison test. (**E**) NOD islets were stimulated with IL-17F or IL-17A for 4 h and *Nfkbiz* expression measured by Taqman quantitative RT-PCR (n = 3 for all groups), p < 0.001 (***) One-Way ANOVA with Bonferonni’s Multiple Comparison test. (**F**) IL-17RA knockout (left panel) and RC knockout (right panel) NIT-1 cells were generated with CRISPR/Cas9 and stained with antibodies against IL-17RA (left panel) and RC (right panel) (black = parental control cell line, grey = receptor knockout cell line, light grey = unstained control). (**E**) Wildtype, IL-17RA KO and IL-17RC KO NIT-1 cells were stimulated with IL-17F or IL-17A for 2 h and Nfkbiz expression measured by Taqman quantitative RT-PCR (n = 2 for all groups), p < 0.05 (*), p < 0.01 (**) Two-Way ANOVA with Bonferonni’s Multiple Comparison test.

To define the expression of the five IL-17R family members (IL-17RA-E) in β-cells, we quantified receptor mRNA levels by qPCR in two mouse β-cell lines (Min6 and NIT-1), NOD islets, and NOD colon tissue, which served as a positive control for all five IL-17R transcripts (Fig. 1A). These data indicate that Min6 and NIT-1 cells express IL-17RA, -RC and -RD (Fig. 1A) but not IL-17RB or -E. Flow cytometry confirmed cell surface expression of IL-17RA, -RC and -RD on NIT-1 cells and primary NOD islets (Fig. 1B).

Expression of the IL-17RA/IL-17RC receptor complex implies that β-cells should respond to both IL-17F and IL-17A^24^. To test for IL-17F sensitivity we stimulated NIT-1, Min6 and primary mouse islets with IL-17F or IL-17A and quantified the expression of *Nfkbiz*, an IL-17A induced transcript^24^. IL-17F induced a significant upregulation of *Nfkbiz* transcript expression in NIT-1 (Fig. 1C), Min6 (Fig. 1D) and mouse islets (Fig. 1E) indicating the β-cells are sensitive to IL-17F. The magnitude of *Nfkbiz* transcript expression in response to IL-17F was increased in primary islets compared to the β-cell lines. This could indicate that IL-17F also acts on other endocrine cells in islets or reflect differences in primary islets compared to cultured β-cell lines. We then generated IL-17RA and -RC deficient NIT-1 cells and validated these by flow cytometry (Fig. 1F). NIT-1 cells were rendered insensitive to IL-17F and IL-17A signals by inactivation of IL-17RA or -RC, as shown by the lack of IL-17F and IL-17A induced *Nfkbiz* in the absence of IL-17RA or -RC (Fig. 1G). These data demonstrate that pancreatic β-cells are responsive to IL-17F via the IL-17RA and -RC subunits.

### IL-17F induces chemokine expression in Min6 cells and primary mouse islets

**Figure 2:**
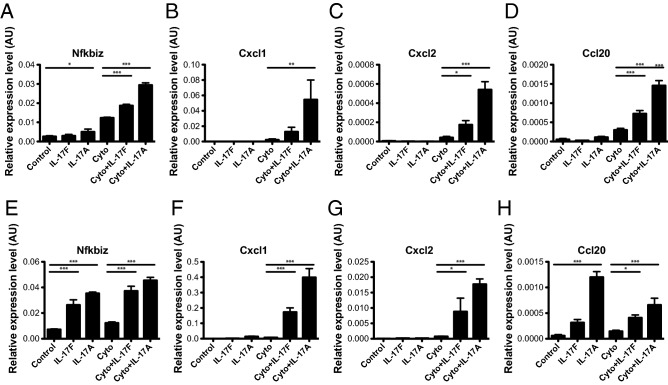
IL-17F induces chemokine expression in mouse β-cell lines and islets. Min6 cells were stimulated with IL-17F or IL-17A in the presence of TNFα + IFNγ (Cyto) for 24 h and (**A**) *Nfkbiz*, (**B**) *Cxcl1*, (**C**) *Cxcl2*, (**D**) *Ccl20* expression measured by Taqman quantitative RT-PCR (n = 4 for all groups), p < 0.05 (*), p < 0.01 (**), p < 0.001 (***) One-Way ANOVA with Bonferonni’s Multiple Comparison test. NOD islets were stimulated with IL-17F or IL-17A in the presence of TNFα + IFNγ (Cyto) for 24 h and (**E**) *Nfkbiz*, (**F**) *Cxcl1*, (**G**) *Cxcl2*, (**H**) *Ccl20* expression measured by Taqman quantitative RT-PCR (n = 6 for all groups), p < 0.05 (*), p < 0.001 (***) One-Way ANOVA with Bonferonni’s Multiple Comparison test.

Previous studies show that IL-17A induces the expression of inflammatory chemokines in mouse and human islets^36^. To determine whether IL-17F exhibits similar activities we stimulated Min6 cells with IL-17F alone or in combination with TNFα + IFNγ (Cyto) for 24 h and quantified chemokine expression with qPCR. IL-17F stimulation induced upregulation of the positive control transcript *Nfkbiz* in combination with TNFα + IFNγ, similarly to IL-17A (Fig. 2A). IL-17F also induced the expression of *Cxcl2* and *Ccl20* in combination with TNFα + IFNγ (Fig. 2C,D) and there was a trend toward increased *Cxcl1* that did not reach statistical significance (Fig. 2B). To confirm these findings in primary islets, we next stimulated NOD islets with IL-17F alone or in combination TNFα + IFNγ (Cyto) for 24 h. IL-17F induced upregulation *Nfkbiz* alone and in combination TNFα + IFNγ (Cyto) (Fig. 2E). IL-17F induced upregulation of *Cxcl1* and *Cxcl2* in combination TNFα + IFNγ, while *Ccl20* was upregulated by IL-17F alone and in combination TNFα + IFNγ (Fig. 2H) as has previously been described for IL-17A^36^. Together these data demonstrate that IL-17F induces the expression of chemokine transcripts similarly to IL-17A but with a consistently reduced magnitude of expression.

### IL-17F suppresses the expression of β-cell identity genes

**Figure 3:**
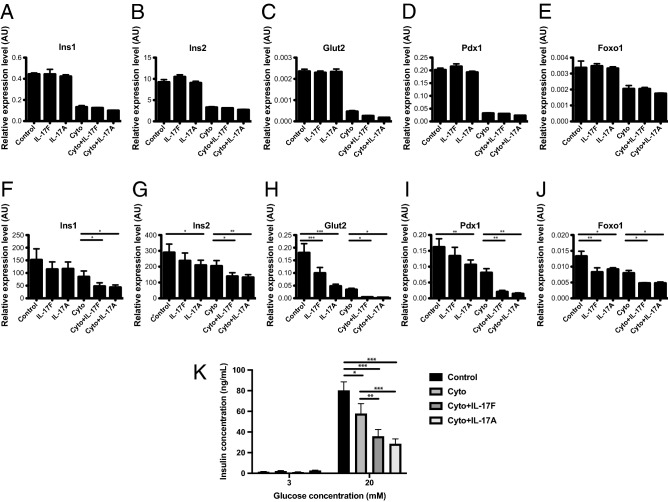
IL-17F suppresses expression of β-cell identity genes and glucose stimulated insulin secretion in mouse islets. Min6 cells were stimulated with IL-17F or IL-17A in the presence of TNFα + IFNγ (Cyto) for 24 h and (**A**) *Ins1*, (**B**) *Ins2*, (**C**) *Glut2*, (**D**) *Pdx1* and (**E**) *Foxo1* expression measured by Taqman quantitative RT-PCR (n = 4 for all groups). NOD islets were stimulated with IL-17F or IL-17A in the presence of TNFα + IFNγ (Cyto) for 24 h and (**F**) *Ins1*, (**G**) *Ins2*, (**H**) *Glut2*, (**I**) *Pdx1* and (**J**) *Foxo1* expression measured by Taqman quantitative RT-PCR (n = 6 for all groups), p < 0.05 (*), p < 0.01 (**), p < 0.001 (***) One-Way ANOVA with Bonferonni’s Multiple Comparison test. (**K**) NOD islets were stimulated with IL-17F or IL-17A in the presence of TNFα + IFNγ (Cyto) for 48 h and glucose stimulated insulin secretion assay performed (3 independent experiments, n = 4 for all groups), p < 0.05 (*), p < 0.01 (**), p < 0.001 (***) Two-Way ANOVA with Bonferonni’s Multiple Comparison test.

Inflammatory cytokines suppress the expression of β-cell identity genes and induce a dysfunctional β-cell state in NOD mice^3,4^, suggesting that this phenomenon may be important in type 1 diabetes pathogenesis. The role of IL-17 family cytokines in these processes have not been tested to date. To determine whether IL-17F or IL-17A could similarly suppress the expression of these β-cell identity genes, we stimulated Min6 cells with IL-17F alone and in combination TNFα + IFNγ (Cyto) for 24 h and quantified mRNA levels of a panel of β-cell identity genes including *Ins1* (Fig. 3A), *Ins2* (Fig. 3B), *Glut2* (Fig. 3C), *Pdx1* (Fig. 3D) and *Foxo1* (Fig. 3E)^37,38^. We observed substantial suppression of expression for *Ins1*, *Ins2*, *Glut2*, *Pdx1* and *Foxo1* in response to TNFα + IFNγ, in accordance with previous studies^3^. While we observed slight decreases in the expression of the transcript panel in response to IL-17A in combination with TNFα + IFNγ, these changes were not statistically significant. There was no discernible effect of IL-17F and IL-17A stimulation alone in Min6 cells (Fig. 3A–E).

To determine whether IL-17F or IL-17A may effect the expression of these genes in primary mouse islets, we next stimulated NOD islets with IL-17F or IL-17A alone or in combination with TNFα + IFNγ (Cyto) and quantified the same panel of β-cell identity genes. In these experiments we observed reduced expression across all measured transcripts, *Ins1* (Fig. 3F), *Ins2* (Fig. 3G), *Glut2* (Fig. 3H), *Pdx1* (Fig. 3I), and *Foxo1* (Fig. 3J) in response to IL-17F or IL-17A in combination with TNFα + IFNγ. In addition IL-17A alone reduced the expression of *Ins2* (Fig. 3G), *Glut2* (Fig. 3H), *Pdx1* (Fig. 3I), and *Foxo1* (Fig. 3J) indicating that IL-17A has potent effects in isolation. IL-17F showed similar activities in isolation for *Glut2* (Fig. 3H) and *Foxo1* (Fig. 3J). Together these data indicate that IL-17F and IL-17A potently suppress the expression of β-cell identity genes in combination with TNFα + IFNγ in primary mouse islets, while there was evidence that IL-17A, and to a lesser extent IL-17F, could also suppress expression of these genes in the absence of TNFα + IFNγ. To determine whether these IL-17F and IL-17A induced changes in the expression of β-cell identity genes resulted in altered β-cell function, we performed glucose stimulated insulin secretion (GSIS) assays under similar conditions. These experiments demonstrated reduced insulin secretion after stimulation with TNFα + IFNγ (Cyto) (Fig. 3K) and we observed further reductions in insulin secretion in combination either IL-17F or IL-17A. Together these data indicate that IL-17F and IL-17A suppress the expression of β-cell identity genes and impair GSIS in NOD islets consistent with the induction of a dysfunction state.

### IL-17F induces cell death in primary mouse islets

**Figure 4:**
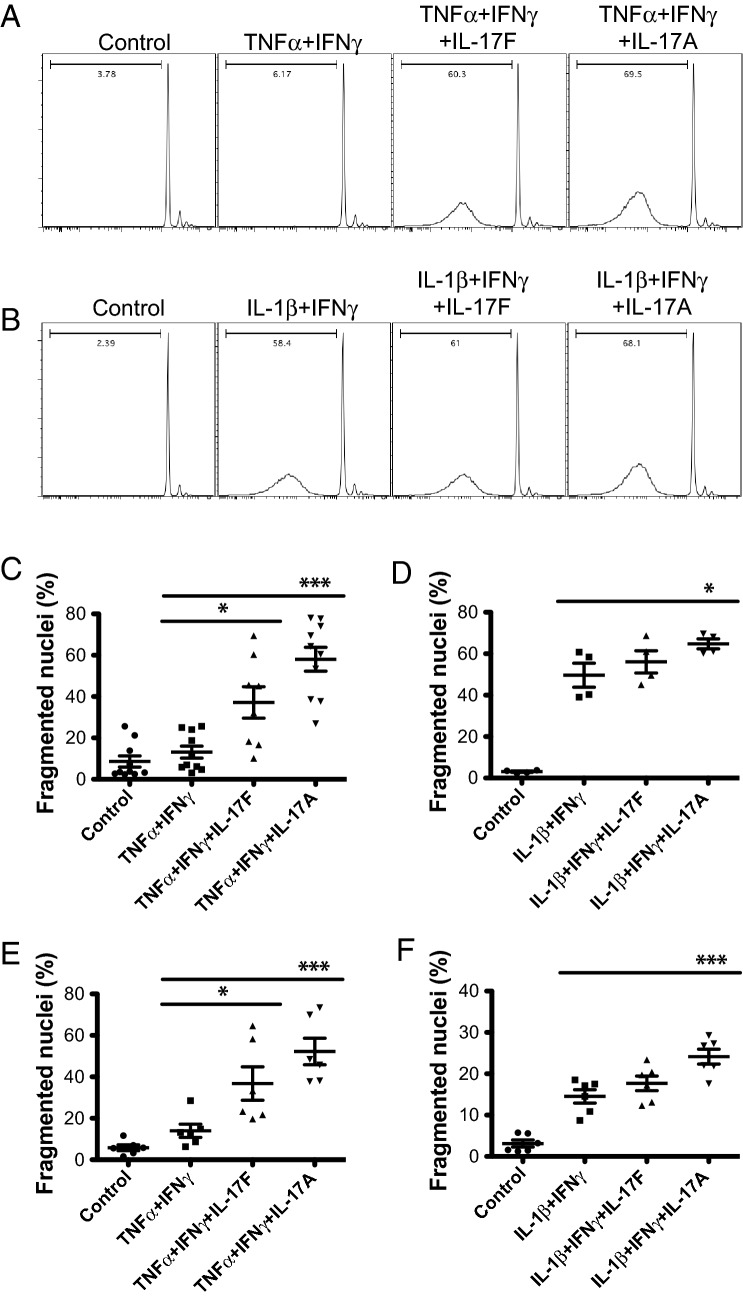
IL-17F induces cell death in mouse islets. NOD islets were stimulated with IL-17F or IL-17A in the presence of (**A**,**C**) TNFα + IFNγ or (**B**,**D**) IL-1β + IFNγ for 4 days and cell death quantified by DNA fragmentation (n = 5 for TNFα + IFNγ and n = 2 for IL-1β + IFNγ), p < 0.05 (*), p < 0.001 (***) One-Way ANOVA with Bonferonni’s Multiple Comparison test. (**C**) C57Bl/6 islets were stimulated with IL-17F or IL-17A in the presence of (**C**) TNFα + IFNγ or (**D**) IL-1β + IFNγ for 4 days and cell death quantified by DNA fragmentation (n = 3 for all groups), p < 0.05 (*), p < 0.001 (***) One-Way ANOVA with Bonferonni’s Multiple Comparison test.

Another previously reported activity of IL-17A is the activation of apoptotic cell death in mouse and human islets^15,36,39^. To test whether IL-17F was also able to induce cell death in primary mouse islets we stimulated NOD islets with IL-17F or IL-17A alone or in combination with inflammatory cytokines and quantified cell death after 4 days by DNA fragmentation. IL-17F or IL-17A did not increase cell death in NOD islets above baseline, as previously described for IL-17A (data not shown)^36^. In contrast, when NOD islets were stimulated with IL-17F, in combination with TNFα + IFNγ, we observed a significant increase in islet cell death similar to IL-17A (Fig. 4A). When islets were stimulated with IL-17F or IL-17A in combination with IL-1β + IFNγ, we also observed increased cell death with IL-17A and a trend towards an increase with IL-17F that did not reach statistical significance (Fig. 4B). Overall the magnitude of the IL-17F and IL-17A induced effects were reduced under these conditions. This may be due to increased baseline levels of cell death induced by IL-1β + IFNγ compared with TNFα + IFNγ or may reflect some level of redundancy between IL-1β and IL-17A/F signalling for activation of cell death.

To determine whether the observed proapoptotic effects of IL-17F were independent of genetic background we performed similar experiments in C57Bl/6 islets (Fig. 4C) When C57Bl/6 islets were stimulated with IL-17F, in combination with TNFα + IFNγ, we again observed a significant increase in islet cell death similarly to IL-17A (Fig. 4C). We also observed increased islet cell death with IL-17A in combination with IL-1β + IFNγ, and a trend towards an increase with IL-17F that did not reach statistical significance (Fig. 4D). Together these data indicate that IL-17F can induce cell death in primary mouse islets in combination with inflammatory cytokines in a similar manner to IL-17A.

### IL-17F induced cell death is iNOS dependent

**Figure 5:**
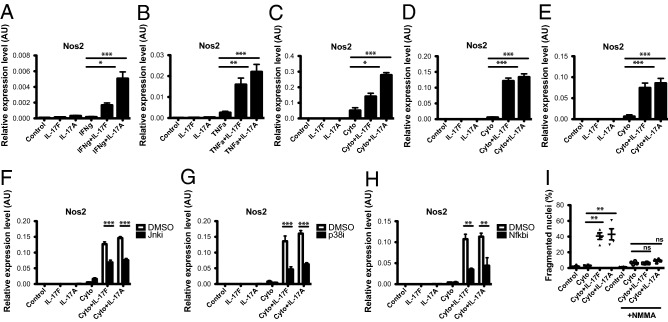
IL-17F induced cell death is iNOS dependent in mouse islets. NOD islets were stimulated with IL-17F or IL-17A in the presence of (**A**) IFNγ or (**B**) TNFα for 4 h and *Nos2* expression measured by Taqman quantitative RT-PCR (n = 5 for all groups), p < 0.05 (*), p < 0.01 (**) One-Way ANOVA with Bonferonni’s Multiple Comparison test. (**C**) Min6 cells, (**D**) NOD islets and (**E**) C57Bl/6 islets were stimulated with IL-17F or IL-17A in the presence of TNFα + IFNγ (Cyto) for 24 h and *Nos2* expression measured by Taqman quantitative RT-PCR (n = 4 for Min6 cells and C57Bl/6 islets, n = 6 for NOD islets), p < 0.05 (*), p < 0.001 (***) One-Way ANOVA with Bonferonni’s Multiple Comparison test. (**F**) NOD islets were stimulated with IL-17F or IL-17A and TNFα + IFNγ (Cyto) +/− Jnk inhibitor (SP600125) at 50 μM for 24 h and *Nos2* expression measured by Taqman quantitative RT-PCR (n = 2 for all groups), p < 0.001 (***) Two-Way ANOVA with Bonferonni’s Multiple Comparison test. (**G**) NOD islets were stimulated with IL-17F or IL-17A and TNFα + IFNγ (Cyto) ± p38 inhibitor (SB203580) at 20 μM for 24 h and *Nos2* expression measured by Taqman quantitative RT-PCR (n = 2 for all groups), p < 0.001 (***) Two-Way ANOVA with Bonferonni’s Multiple Comparison test. (**H**) NOD islets were stimulated with IL-17F or IL-17A and TNFα + IFNγ (Cyto) ± NF-κB inhibitor (BAY11-7082) at 10 μM for 24 h and *Nos2* expression measured by Taqman quantitative RT-PCR (n = 2 for all groups), p < 0.01 (**) Two-Way ANOVA with Bonferonni’s Multiple Comparison test. (**I**) NOD islets were stimulated with IL-17F or IL-17A in the presence of TNFα + IFNγ (Cyto) for 4 days ± NMMA (1 mM) and cell death quantified by DNA fragmentation (n = 2 for all groups), p < 0.01 (**) One-Way ANOVA with Bonferonni’s Multiple Comparison test.

Previous reports have demonstrated that IL-17A induced cell death is iNOS dependent in mouse islets^15,39^. To test whether IL-17F induced islet cell death occurred via similar mechanisms we first tested whether IL-17F was able to induce *Nos2* expression. We stimulated NOD islets with IL-17F in the presence of IFNγ and/or TNFα for 24 h and *Nos2* mRNA expression quantified by Taqman qPCR. While IL-17F alone did not induce *Nos2* expression in NOD islets, IL-17F induced a significant increase in *Nos2* expression in NOD islets in combination with IFNγ (Fig. 5A) or TNFα (Fig. 5B). Further in combination with TNFα + IFNγ we observed a significant induction of *Nos2* in response to IL-17F in Min6 cells (Fig. 5C), NOD islets (Fig. 5D) and C57Bl/6 islets (Fig. 5E).

We next tested the requirement of downstream signalling pathways for IL-17F induction of *Nos2*. Since IL-17RA and -RC engagement activates MAPK and NF-κB signalling^24^, we tested the effect of pharmacological inhibitors of the JNK, p38 and NF-κB pathways on IL-17F induced *Nos2* expression. NOD islets were stimulated with IL-17F in combination with TNFα + IFNγ for 24 h in the presence of inhibitors. IL-17F induced expression was similarly reduced with a Jnk inhibitor (SP600125, Fig. 5F), p38 inhibitor (SB203580, Fig. 5G) or an NF-κB inhibitor (BAY11-7082, Fig. 5H). Finally to test whether IL-17F induced cell death was iNOS dependent we performed islet cell death assays in the presence of the iNOS inhibitor *N*-methylarginine (NMMA). In this context IL-17F induced cell death was completely ablated in the presence of NMMA, indicating that IL-17F induced islet cell death is critically dependent upon iNOS (Fig. 5I). These data indicate that IL-17F induces *Nos2* expression in mouse β-cell lines and primary mouse islets in combination with inflammatory cytokines resulting in increased levels of cell death.

## Discussion

Our studies identify novel pathogenic functions for IL-17F in mouse pancreatic β-cells. We show that IL-17F signals via the IL-17RA and -RC receptor subunits and causes deleterious effects in islets including expression of inflammatory chemokines, suppression of β-cell identity gene expression and insulin secretion and induction of cell death, all of which could contribute to the progressive loss of functional β-cell mass in type 1 diabetes. The effects of IL-17F were similar to IL-17A for the majority of parameters measured, but with a generally reduced magnitude of expression induced by IL-17F, which is a phenomenon that has been observed in other experimental systems^27,40^. These data suggest that IL-17F and IL-17A are likely to exhibit some level of functional redundancy in β-cells. This is in keeping with other studies demonstrating overlapping functions of IL-17F and IL-17A in models of autoimmunity and pathogen clearance^26,27^. Further studies are required to determine the full extent of overlapping and/or divergent functions of IL-17F and IL-17A in pancreatic β-cells.

The relative importance of type 17 immune responses for the development of type 1 diabetes in animal models and human patients remains unclear. One major issue is the conflicting findings regarding the function of IL-17A. While IL-17A has pathogenic effects on β-cells^11–15^ and anti-IL-17A antibody treatment protects mice from type 1 diabetes^20^, IL-17A gene deficient and shRNA knockdown NOD mice have essentially normal kinetics of disease onset^22,23^. These data indicate that IL-17A possesses pathogenic activities but that these are not essential for type 1 diabetes development in mouse models. Our observations that IL-17F exhibits similar pathogenic properties to IL-17A provides some novel insight into this issue and suggests two potential scenarios. Firstly that IL-17F and IL-17A could be functionally redundant in type 1 diabetes and that both genes must be inactivated or inhibited for any potential type 1 diabetes protection to eventuate. Alternatively IL-17F may have a more important role in type 1 diabetes pathogenesis than IL-17A. Both of these scenarios are supported in part by the observation that IL-17A blocking antibodies protect NOD mice from type 1 diabetes^20^, since these antibodies block both IL-17A homodimers and IL-17A:IL-17F heterodimers^41^, and will impinge upon IL-17F function. Thus we speculate that IL-17F deficient or IL-17F/IL-17A doubly deficient NOD mice may exhibit more protection from type 1 diabetes than IL-17A deficient NOD. Similarly, interventions that block IL-17F and IL-17A simultaneously may have improved therapeutic utility in type 1 diabetes compared to interventions that block either cytokine alone.

In summary our studies provide the first evidence that IL-17F has pathogenic activities in mouse pancreatic β-cells. These data indicate that it will be important to consider the function of IL-17F alongside IL-17A when evaluating the effect of IL-17 cytokines in type 1 diabetes and that further study of IL-17F is required to more fully elucidate the type 17 dependent pathogenic mechanisms in type 1 diabetes.

## Supplementary information

Supplementary Information.
