# Supplementary material for: IL-17F induces inflammation, dysfunction and cell death in mouse islets
Source: Sci Rep. 2020 Aug 4;10:13077. doi: 10.1038/s41598-020-69805-2 (PMC7403586; doi:10.1038/s41598-020-69805-2)
Supplement: Supplementary file 1 — Supplementary Information. [file 41598_2020_69805_MOESM1_ESM.pdf]

IL-17F induces inflammation, dysfunction and cell death in mouse islets

(IL-17F in mouse islets)

Tara Catterall[1], Stacey Fynch[1], Thomas W. H. Kay[1,2], Helen E. Thomas[1,2], Andrew P. R. Sutherland[1,2]

[1]St Vincent's Institute of Medical Research, Melbourne, Australia

[2]The University of Melbourne, Department of Medicine, St. Vincent's Hospital, Fitzroy, Victoria, Australia

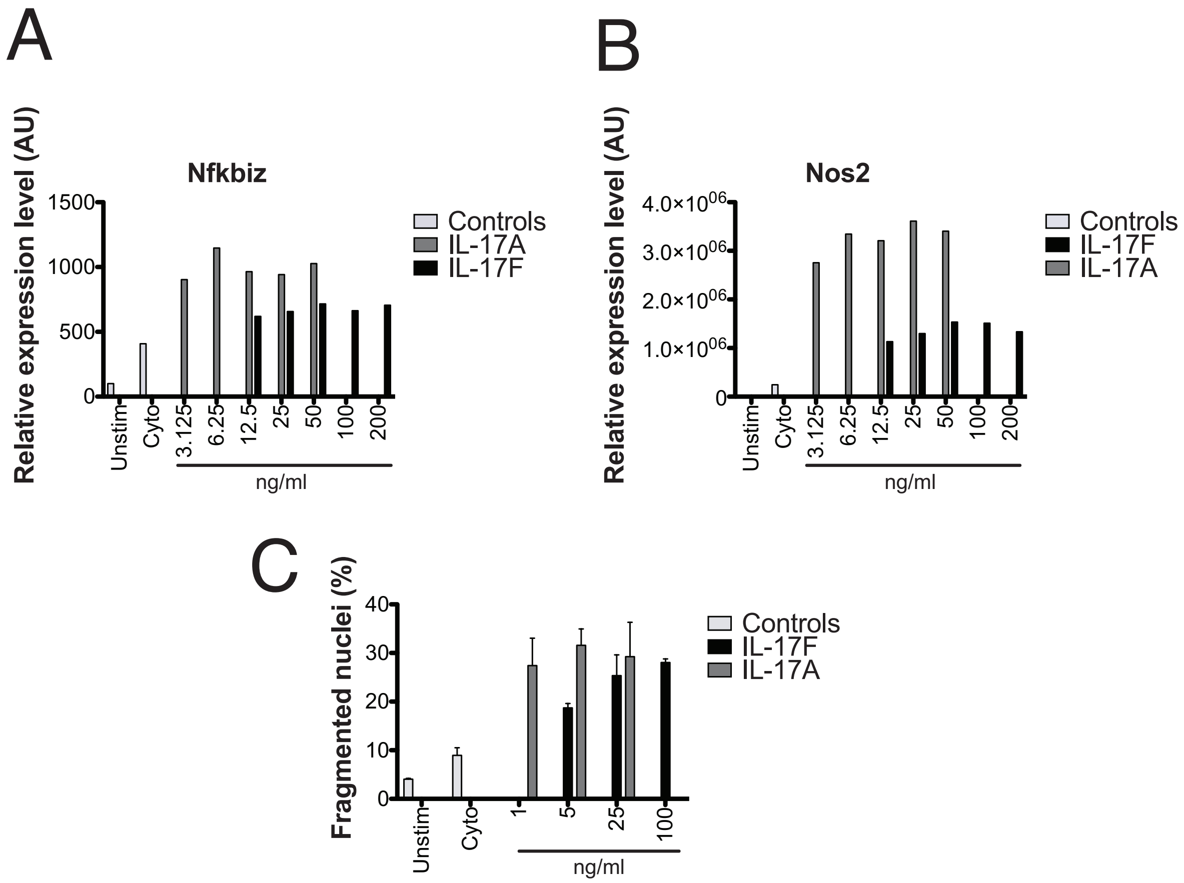

<u>Supplementary Figure 1:</u> ***Titration of IL-17F and IL-17A induced effects in MIN6 cells and primary islets.***

A) MIN6 cells were stimulated with IL-17F or IL-17A at various concentrations for 4 hrs and *Nfkbiz* expression measured by Taqman quantitative RT-PCR. B) MIN6 cells were stimulated with IL-17F or IL-17A at various concentrations for 24 hrs in combination with TNFα+IFNγ and *Nos2* expression measured by Taqman quantitative RT-PCR. C) NOD islets were stimulated with IL-17F or IL-17A at various concentrations in combination with TNFα+IFNγ and cell death quantified by DNA fragmentation.

Gene	gRNA #		Primer A	Primer B
IL-17RA	1	5' of exon	5'-CACCGAGCATTAGAAAAGAGCAGGC-3'	5'-AAACGCCTGCTCTTTTCTAATGCTC-3'
	2	3' of exon	5'-CACCGCTTAGGAACACAGTGCAGTG-3'	5'-AAACCACTGCACTGTGTTCCTAAGC-3'
IL-17RC	1	5' of exon	5'-CACCGGAAGCTCCCCTGCCCTCTGC-3'	5'-AAACGCAGAGGGCAGGGGAGCTTCC-3'
	2	3' of exon	5'-CACCGAGGTGGCTCTTCTCCTCAGG-3'	5'-AAACCCTGAGGAGAAGAGCCACCTC-3'

Supplementary Table 1: List of primers for CRISPR/Cas9 gene editing

Gene	Taqman Probe set #
Actb	Mm02619580_g1
Cxcl1	Mm04207460_m1
Cxcl2	Mm00436450_m1
Ccl20	Mm04207460_m1
Foxo1	Mm00490671_m1
Glut2	Mm00446229_m1
Il17ra	Mm00434214_m1
Il17rb	Mm00444709_m1
Il17rc	Mm00506606_m1
Il17rd	Mm00460340_m1
Il17re	Mm01189488_m1
Ins1	Mm01950294_s1
Ins2	Mm00731595_gH
Nfkbiz	Mm00600522_m1
Nos2	Mm00440502_m1
Pdx1	Mm00435565_m1

Supplementary Table 2: List of primers for Taqman quantitative RT-PCR
